# Association of Esophageal Inflammation, Obesity and Gastroesophageal Reflux Disease: From FDG PET/CT Perspective

**DOI:** 10.1371/journal.pone.0092001

**Published:** 2014-03-18

**Authors:** Yen-Wen Wu, Ping-Huei Tseng, Yi-Chia Lee, Shan-Ying Wang, Han-Mo Chiu, Chia-Hung Tu, Hsiu-Po Wang, Jaw-Town Lin, Ming-Shiang Wu, Wei-Shiung Yang

**Affiliations:** 1 Department of Internal Medicine, National Taiwan University Hospital, Taipei, Taiwan; 2 Department of Nuclear Medicine, National Taiwan University Hospital, Taipei, Taiwan; 3 Department of Nuclear Medicine and Cardiology Division of Cardiovascular Medical Center, Far Eastern Memorial Hospital, New Taipei City, Taiwan; 4 National Yang-Ming University School of Medicine, Taipei, Taiwan; 5 Graduate Institute of Clinical Medicine, College of Medicine, National Taiwan University, Taipei, Taiwan; 6 Research Center for Developmental Biology and Regenerative Medicine, National Taiwan University, Taipei, Taiwan; 7 Graduate Institute of Epidemiology and Preventive Medicine, College of Public Health, National Taiwan University, Taipei, Taiwan; 8 School of Medicine, Fu Jen Catholic University, New Taipei City, Taiwan; 9 Department of Internal Medicine, E-Da Hospital, Kaohsiung, Taiwan; University Hospital Llandough, United Kingdom

## Abstract

**Objective:**

Gastroesophageal reflux disease (GERD) is associated with bothersome symptoms and neoplastic progression into Barrett's esophagus and esophageal adenocarcinoma. We aim to determine the correlation between GERD, esophageal inflammation and obesity with ^18^F-Fluorodeoxyglucose (FDG) positron emission tomography/computed tomography (PET/CT).

**Methods:**

We studied 458 subjects who underwent a comprehensive health check-up, which included an upper gastrointestinal endoscopy, FDG PET/CT and complete anthropometric measures. GERD symptoms were evaluated with Reflux Disease Questionnaire. Endoscopically erosive esophagitis was scored using the Los Angeles classification system. Inflammatory activity, represented by standardized uptake values (SUV_max_) of FDG at pre-determined locations of esophagus, stomach and duodenum, were compared. Association between erosive esophagitis, FDG activity and anthropometric evaluation, including body mass index (BMI), waist circumference, visceral and subcutaneous adipose tissue volumes were analyzed.

**Results:**

Subjects with erosive esophagitis (n = 178, 38.9%) had significantly higher SUV_max_ at middle esophagus (2.69±0.74 vs. 2.41±0.57, *P*<.001) and esophagogastric junction (3.10±0.89 vs. 2.38±0.57, *P*<.001), marginally higher at upper esophageal sphincter (2.29±0.42 vs. 2.21±0.48, *P* = .062), but not in stomach or duodenum. The severity of erosive esophagitis correlated with SUV_max_ and subjects with Barrett's esophagus had the highest SUV_max_ at middle esophagus and esophagogastric junction. Heartburn positively correlated with higher SUV_max_ at middle oesophagus (*r* = .262, *P* = .003). Using multivariate regression analyses, age (*P* = .027), total cholesterol level (*P* = .003), alcohol drinking (*P* = .03), subcutaneous adipose tissue (*P*<.001), BMI (*P*<.001) and waist circumference (*P*<.001) were independently associated with higher SUV_max_ at respective esophageal locations.

**Conclusions:**

Esophageal inflammation demonstrated by FDG PET/CT correlates with endoscopic findings and symptomatology of GERD. Obesity markers, both visceral and general, are independent determinants of esophageal inflammation.

## Introduction

The incidence and prevalence of gastroesophageal reflux disease (GERD) have increased remarkably worldwide over the past decades, partly related to the epidemics of obesity and metabolic syndrome [1], [2]. GERD has been associated with a broad spectrum of symptoms and has a great impact on the quality of life of patients [3]. Moreover, long-standing gastroesophageal reflux has been associated with the development of Barrett's esophagus, which poses an increased risk of esophageal adenocarcinoma [4], [5]. Chronic mucosa damage by the refluxate is thought to stimulate the inflammatory and proliferative responses in the esophageal squamous epithelium [6]. Recently, obesity has been found to be a strong risk factor for developing GERD-related symptoms and complications [7], [8]. In addition to increasing intra-abdominal pressure, visceral adipose tissue produces multiple adipokines and proinflammatory cytokines, which may result in low grade chronic inflammation and further promote neoplastic progression [9], [10].

^18^F-Fluorodeoxyglucose (FDG) positron emission tomography/computed tomography (PET/CT) assesses not only anatomical structures, but also the degree of local glucose metabolism and recently has been proposed as a promising tool in the evaluation of non-neoplastic diseases, such as inflammatory and infectious diseases [11]–[15]. In Taiwan, the incidence of *Helicobacter pylori-*related upper gastrointestinal (GI) pathologies remain high, and self-paid health examinations, including a complete metabolic profile as well as both upper endoscopy and PET-CT, are widely available to the general population. This provides us a unique opportunity to explore the complex relationship of GERD, esophageal inflammation and obesity. Therefore, through analyzing subjects who have undergone both an upper GI endoscopy and PET/CT as part of a comprehensive health examination in our institute, we aim to determine whether extent of esophageal inflammation, as shown by the FDG uptake on PET/CT, correlates with the severity of erosive reflux disease on endoscopy, as well as the reflux symptoms. In addition, with the help of concurrent low dose CT scan, we quantitatively determined the volume of visceral and subcutaneous adipose tissue and we aim to assess the correlation between abdominal obesity and esophageal inflammation of GERD.

## Materials and Methods

### Ethics Statement

This study was approved by the Ethical Committee of National Taiwan University Hospital (No. 201204030RIB). Data from the prospectively established cohort who have voluntarily participated in a self-paid health check-up program at the Health Management Center of National Taiwan University Hospital were accessed; all subjects have provided written inform consent before the program. Attendees of health check-up examinations in our institute were from the general population. Such an examination fee was generally affordable with approximate 1/30 of the gross national income per capita in Taiwan that the participants did not belong to any particular socio-economic class or share a unifying form of employment, and were recruited through advertising messages for health-promotion purposes.

### Study protocol

In this health check-up program, PET/CT were optional and under the discretion of each subject. Therefore, consecutive subjects who have undergone both an upper GI endoscopy and PET/CT as part of this program between January 2004 and June 2011 were included for the analysis. Those who had a history of previous GI surgery or had a history of gastroesophageal malignancy were excluded from this study. The standard protocol consisted of a self-administered questionnaire, interview by an internal medicine physician, physical examination, blood biochemical tests, plain radiography, abdominal ultrasonography, ^13^C urea breath test for *Helicobacter pylori* infection, and endoscopy. Insulin resistance was measured based on the Homeostasis Model of Assessment-Insulin Resistance [16].

Prior to examination, all subjects filled out a standard questionnaire that collected demographic information, symptoms involving all body systems in the past 3 months, medical and medication history, and social habits (smoking and alcohol). From Jan 2010, we further incorporated a validated Reflux Disease Questionnaire (RDQ) to evaluate the gastroesophageal reflux symptoms [17]. RDQ comprises 12 questions assessing the frequency and severity of three subscales of heartburn, regurgitation and dyspepsia. All questions were scored on a Likert scale with scores ranging from 0 to 5 for frequency (not present to daily) and severity (not present to severe). The presence of each related symptom was verified by internal medicine consultation. We defined asymptomatic subjects as those with RDQ score  = 0 and symptomatic subjects as those with RDQ score ≥ 1.

### Endoscopic Examination

All endoscopic procedures were performed by experienced endoscopists using a GIF 240 or GIF 260 videoendoscope (Olympus, Tokyo, Japan).The reliability of endoscopic evaluation of erosive esophagitis and Barrett's esophagus has been confirmed [18], [19]. The esophagus was carefully evaluated, and all endoscopic findings were meticulously recorded and stored in a computerized database. Erosive esophagitis was scored using the Los Angeles classification system with standard comparator photos [20]. Barrett's esophagus was confirmed by histological identification of specialized columnar epithelium with intestinal metaplasia. Hiatal hernia was defined as a distance of at least 2 cm between the esophagogastric junction and the diaphragmatic hiatus. Subjects who were found to have esophagitis, Barrett's esophagus or other esophageal neoplasms would be referred to their primary care physicians or specialists for further evaluation and treatment.

### PET/CT Imaging and Analysis

PET/CT examination was performed within one week of other health examinations. All PET/CT studies were performed on a hybrid PET/CT scanner (Discovery LS, General Electric Medical Systems, Milwaukee, WI, USA), combining a GE Advance NXi PET scanner and a 16-slice helical multi-detector CT scanner (Light Speed Plus). Each subject fasted for at least 8 hours, and underwent PET/CT scans from the vertex of the skull to the proximal thighs in 2-dimensional (2D) mode at 60 min after intravenous administration of FDG (6 MBq [0.162 mCi]/kg body weight). The blood glucose measurements before FDG injection were less than 115 mg/dl in all patients. A low-dose whole-body CT scan for attenuation correction and anatomical localization of the PET signal was performed. PET and CT data were transformed into DICOM format, and sent to a workstation (Xeleris Functional Imaging Station, GE) for 3D post-processing, coregistration, fusion, and separate review.

The PET/CT scans were read by 2 experienced reviewers (YW Wu and SY Wang) in consensus to determine the localization and the patterns of FDG accumulation in the upper gastrointestinal tract. These reviewers were blinded to the endoscopic findings. A region of interest (ROI) of 3×3 pixels was manually placed on and slid along the 5 index regions, including the upper esophageal sphincter, middle esophagus (retro-cardiac portion), esophagogastric junction, stomach, and duodenum using anatomical landmarks on CT scan. The standardized uptake value (SUV) of FDG was calculated as: (activity in ROI in uCi/mL)/(injected dose in mCi/weight in kg). We recorded the highest SUV (SUV_max_) of each location for subsequent analysis. Focality of FDG uptake, which combines intensity and length of the lesion in 1 complementary parameter, was determined as described by Roedl et al. with slight modifications. [21] In brief, the presence of focal uptake was defined as <3 cm in length and intensity score >0 (closer to brain than to liver uptake).

Abdominal adiposity was assessed with an offline workstation (Advantage workstation, GE) from the non-enhanced CT raw data. Twenty-five contiguous 5 mm thick slices (120 kVp, 400 mA, gantry rotation time 500 ms, table feed 3∶1) were acquired, covering 125 mm above the level of S1. The raw data were reconstructed using a 55 cm field of view. Subcutaneous fat was defined as the extraperitoneal fat between skin and muscle, with attenuation ranging from −195 to −45 Hounsfield units and a window center of −120 Hounsfield units to identify pixels containing adipose tissue. In order to separate visceral from subcutaneous fat, the abdominal muscular wall separating the two compartments was manually traced. The visceral adipose tissue area (VAT) and subcutaneous adipose tissue area (SAT) were determined by automatic planimetry at the umbilical level. The intra- and inter-reader reproducibility was high for the SAT and VAT measurement (inter-reader and intra-reader comparisons, all *r*≥0.98, p<0.0001) in our laboratory. [15].

### Statistical Analysis

First, we compared basic demographic data, anthropometric measurements, and FDG uptake at index regions between subjects with and without erosive esophagitis. In addition, subjects with erosive esophagitis were further classified into mild (Los Angeles classification grade A or B) and severe esophagitis (grade C or D), and 1-way analysis of variance was used to test for linear trends in SUV_max_ across the severity levels among all subjects. Continuous data were expressed as the mean ± standard deviation (SD) and compared by Student *t* test or non-parametric test, when appropriate. For SUV_max,_ the median and interquartile ranges were also provided. Categorical data were expressed as percentage and analyzed by Pearson χ^2^ tests or Fisher exact tests, as appropriate.

Second, we assessed the determinants of esophageal inflammation in terms of SUV_max_ of FDG on PET/CT. Univariable relationships between SUV_max_ at respective index locations and traditional risk factors of GERD were assessed with *Pearson's* correlation. Here traditional risk factors include age, male gender, lifestyle factors, metabolic factors, *Helicobacter pylori* infection, and patterns of abdominal fat distribution on CT. Since SUV_max_ represents a continuous measure of the severity of esophageal inflammation, linear regression models were used to determine whether these variables were significant predictors of esophageal inflammation. A two-tailed *P* value of <.05 was considered statistically significant. All statistical analyses were performed using SPSS 16 (SPSS, Inc., Chicago, IL, USA).

## Results

### Demographic Characteristics

A total of 458 subjects who underwent the health check-up program were analyzed. Among them, 178 subjects (38.9%) were diagnosed with erosive esophagitis by endoscopy with the mean age of 56 years (range: 30–84 years), and 147 (82.6%) were male. Most cases of erosive esophagitis were mild in severity (120 subjects with grade A, 41 with grade B, 16 with grade C, and 1 with grade D). Six had Barrett's esophagus, and hiatal hernias were found in 19 subjects (10.7%). No esophageal high-grade dysplasia or cancer was found in all study subjects during the index check-up and follow-up. Compared with subjects without endoscopically evident esophagitis, the subjects with erosive esophagitis were male predominant, had higher systolic blood pressure, higher fasting blood glucose, higher HbA1C levels, higher insulin resistance, lower level of high-density lipoprotein, higher body mass index (BMI), larger waist circumference, more visceral adipose tissue volume and less *Helicobacter pylori* infection (Table 1).

**Table 1:** Basic demographics and SUV_max_ of FDG at index upper gastrointestinal locations.

Characteristic	With Erosive Esophagitis (n = 178)	Without Erosive Esophagitis (n = 280)	P*
Age, y	56.0±10.4	54.3±9.9	.079
Male gender	147 (82.6)	176 (62.9)	<.001
Smoking	41 (23.0)	45 (16.1)	.063
Drinking	42 (23.6)	47 (16.8)	.073
SBP, mm Hg	122.1±13.5	118.7±15.0	.011
Fasting blood glucose, mg/dL	101.2±26.8	95.9±20.9	.025
HbA1C, %	5.93±0.90	5.72±0.80	.010
Triglycerides, mg/dL	137.1±74.2	127.4±77.4	.183
Total cholesterol, mg/dL	205.0±36.9	204.0±32.9	.763
HDL, mg/dL	43.9±10.3	46.8±12.1	.008
LDL, mg/dL	121.4±32.1	118.9±29.6	.397
HOMA-IR	2.15±1.92	1.45±1.23	.001
BMI, kg/m^2^	25.7±3.3	24.3±3.3	<.001
Waist circumference, cm	90.7±8.4	87.1±9.6	<.001
Helicobacter pylori infection	39 (25.7)	93 (49.2)	<.001
**Abdominal CT adipose tissue volume, cm^3^**			
Total abdominal adipose tissue	179.3±57.4	169.0±61.7	.075
Visceral adipose tissue	72.8±27.6	61.7±29.0	<.001
Subcutaneous adipose tissue	106.4±41.6	107.3±95.4	.832
**Endoscopic findings**			
Hiatal hernia	19 (10.7)	1 (0.4)	<.001
Barrett's esophagus	6 (3.4)	-	-
EE, LA Grade A+B	161 (90.4)	-	-
EE, LA Grade C+D	17 (9.6)	-	-
**FDG SUV_max_**			
Upper esophageal sphincter	2.29±0.42	2.21±0.48	.062
	(2.3, 2.0–2.6)	(2.2, 1.9–2.5)	
Middle esophagus	2.69±0.74	2.41±0.57	<.001
	(2.6, 2.2–3.1)	(2.4, 2.1–2.8)	
Esophagogastric junction	3.10±0.89	2.38±0.57	<.001
	(2.9, 2.6–3.5)	(2.4, 2.0–2.8)	
Focality at esophagogastric junction	55 (30.9)	17 (6.1)	<.001
Stomach	3.34±1.01	3.30±1.00	.684
	(3.2, 2.7–3.8)	(3.3, 2.6–3.8)	
Duodenum	2.49±0.82	2.53±0.79	.620
	(2.4, 1.8–2.9)	(2.4, 2.0–3.0)	

a. Data are presented as mean ± standard deviation (median, interquartile range) or number (percentage).

b. Abbreviation: FDG, ^18^F-Fluorodeoxyglucose; EE, erosive esophagitis; LA, Los Angeles classification system; SBP, systolic blood pressure; DBP, diastolic blood pressure; HDL, high-density lipoprotein; LDL, low-density lipoprotein; HOMA-IR, Homeostasis Model of Assessment-Insulin Resistance; BMI, body mass index; RDQ, Reflux Disease Questionnaire; SUV_max_, maximum of standardized uptake values.

c. *P<.05, indicates statistical significance.

### Severity of Erosive Esophagitis and SUV_max_ on PET/CT

**Figure 1:**
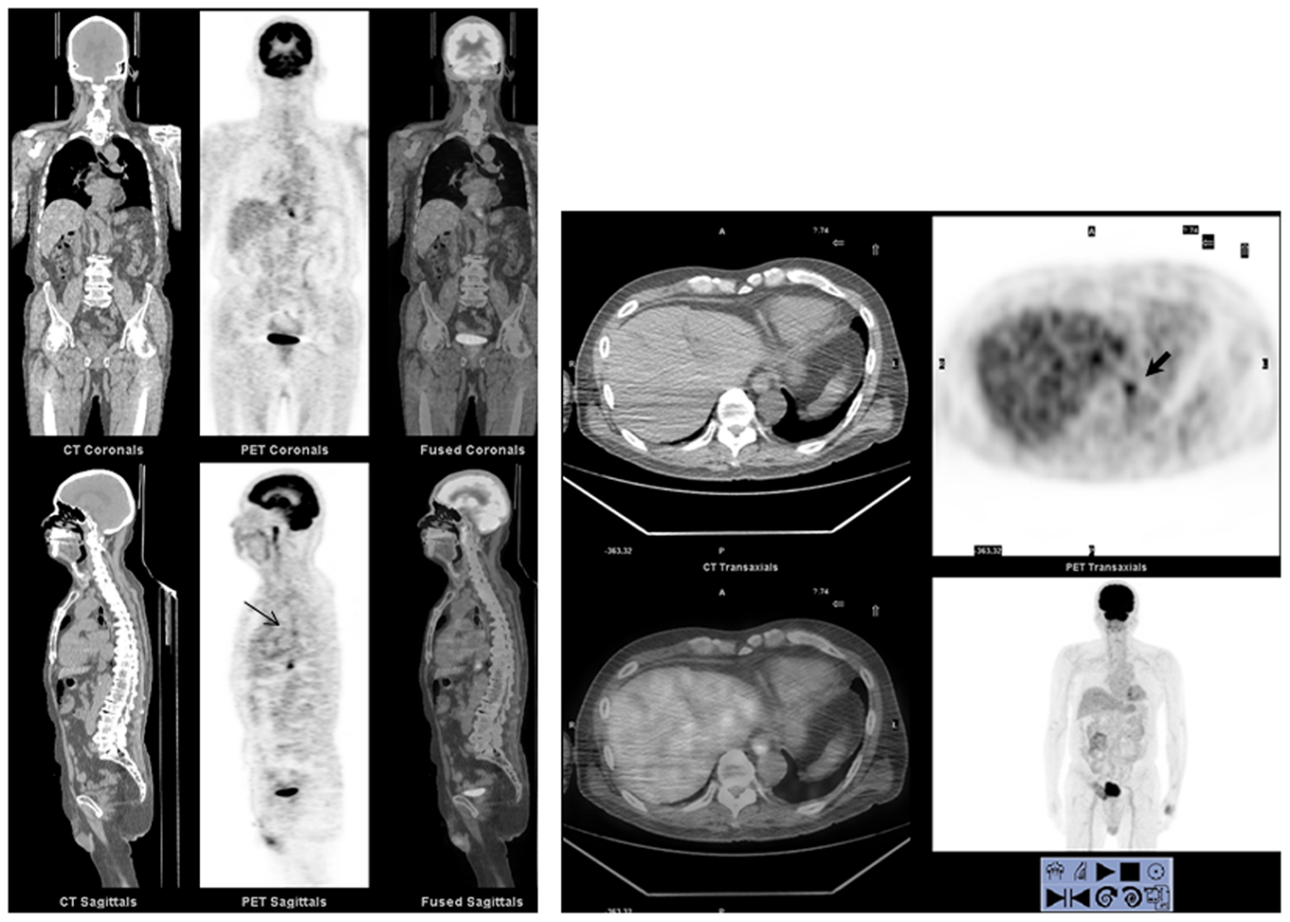
FDG PET/CT images from an 84-year-old male with erosive esophagitis (Los Angeles classification grade A). PET/CT showed two focal areas of FDG accumulation in the middle esophagus (thin arrow on sagittal view, SUV_max_ = 4.5) and at the esophagogastric junction (thick arrows, SUV_max_ = 6.9).

The SUV_max_ at index upper GI locations and the focality pattern of FDG uptake at the esophagogastric junction were analyzed. Six subjects had markedly elevated SUV_max_ at the esophagogastric junction (SUV_max_ >5.5; the highest 6.9) and all had erosive esophagitis (2 subjects with grade A, 2 with grade B, 2 with grade C) and 2 of them also had Barrett's esophagus. Compared with subjects without erosive esophagitis (Table 1), the SUV_max_ in subjects with erosive esophagitis were significantly higher at the middle esophagus (2.69±0.74 (2.6, 2.2–3.1) vs. 2.41±0.57 (2.4, 2.1–2.8), *P*<.001) and esophagogastric junction (3.10±0.89 (2.9, 2.6–3.5) vs. 2.38±0.57 (2.4, 2.0–2.8), *P*<.001), marginally higher at upper esophageal sphincter (2.29±0.42 (2.3, 2.0–2.6) vs. 2.21±0.48 (2.2, 1.9–2.5), *P* = .062), but not in stomach or duodenum. A higher prevalence of focal FDG uptake at the esophagogastric junction was also noted in the erosive esophagitis subjects (30.9% vs. 6.1%, *P*<.001). Representative FDG PET/CT images of erosive esophagitis are shown in Figure 1.

**Figure 2:**
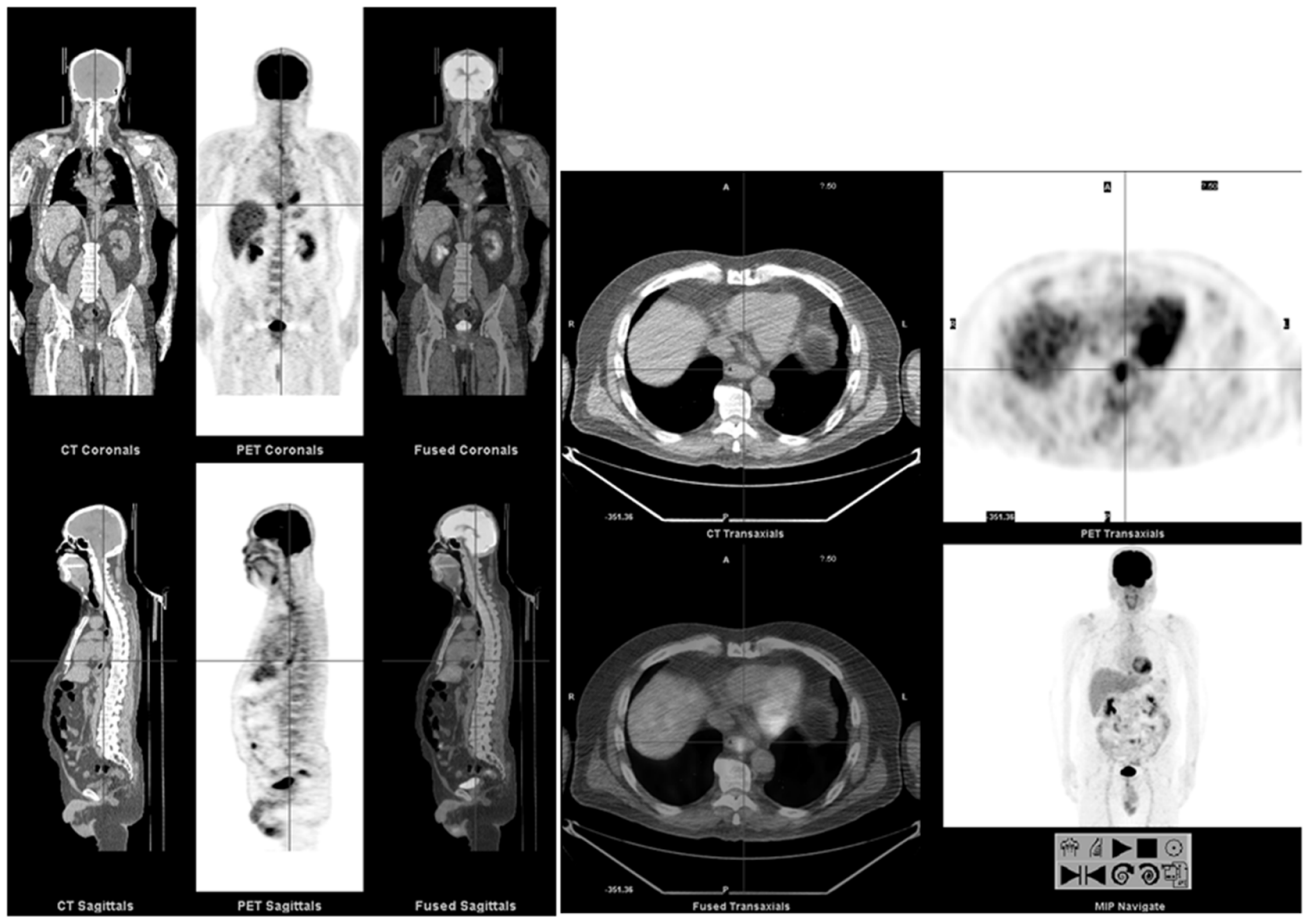
FDG PET/CT images from a 59-year-old male with Barrett's esophagus. PET/CT showed intense FDG accumulation with correlative wall thickening in the esophagogastric junction (cursor, SUV_max_ = 5.6).

We further compared the SUV_max_ in subjects with erosive esophagitis stratified by the esophagitis severity. As shown in Table 2, there was a progressive increase of SUV_max_ in each segment of the esophagus from subjects with no esophagitis to subjects with mild esophagitis and to subjects with severe esophagitis (*P* = .063 for upper esophageal sphincter and *P*<.001 for both middle esophagus and esophagogastric junction). Focal distribution of SUV_max_ at the esophagogastric junction was more frequently observed in subjects with higher grade esophagitis (70.6% vs. 26.7%, *P*<.001). Although the case number was rather small, subjects with Barrett's esophagus had the highest SUV_max_ at middle esophagus (3.18±1.06) and esophagogastric junction (3.95±1.35). Representative FDG PET/CT images of Barrett's esophagus are shown in Figure 2.

**Table 2:** Comparison of SUVmax of FDG at index upper gastrointestinal locations.

	Without Esophagitis	Mild Esophagitis	Severe Esophagitis	*P* *
	(n = 280)	(n = 161)	(n = 17)	
Upper esophageal sphincter	2.21±0.48	2.28±0.42	2.44±0.34	.063
	(2.2, 1.9–2.5)	(2.3, 2.0–2.6)	(2.4, 2.1–2.7)	
Middle esophagus	2.41±0.57	2.66±0.73	2.98±0.75	<.001
	(2.4, 2.1–2.8)	(2.6, 2.2–3.0)	(3.1, 2.3–3.4)	
Esophagogastric junction	2.38±0.57	3.06±0.84	3.47±1.20	<.001
	(2.4, 2.0–2.8)	(2.9, 2.5–3.5)	(3.4, 2.7–3.6)	
Focality at esophagogastric junction	17 (6.1)	43 (26.7)	12 (70.6)	<.001
Stomach	3.30±1.00	3.34±1.00	3.32±1.14	.919
	(3.3, 2.6–3.8)	(3.2, 2.7–3.8)	(3.1, 2.5–3.9)	
Duodenum	2.53±0.79	2.50±0.82	2.37±0.82	.735
	(2.4, 2.0–3.0)	(2.4, 1.9–2.9)	(2.1, 1.7–2.7)	

a. Data are presented as mean ± standard deviation deviation (median, interquartile range) or number (percentage).

b. Abbreviation: FDG, 18F-Fluorodeoxyglucose; SUVmax, maximum of standardized uptake values.

c. Mild esophagitis refers to erosive esophagitis, LA Grade A+B; severe esophagitis refers to erosive esophagitis, LA Grade C+D.

*P<0.05 indicates statistical significance.

### Gastroesophageal Reflux Symptoms and SUV_max_ on PET/CT

**Figure 3:**
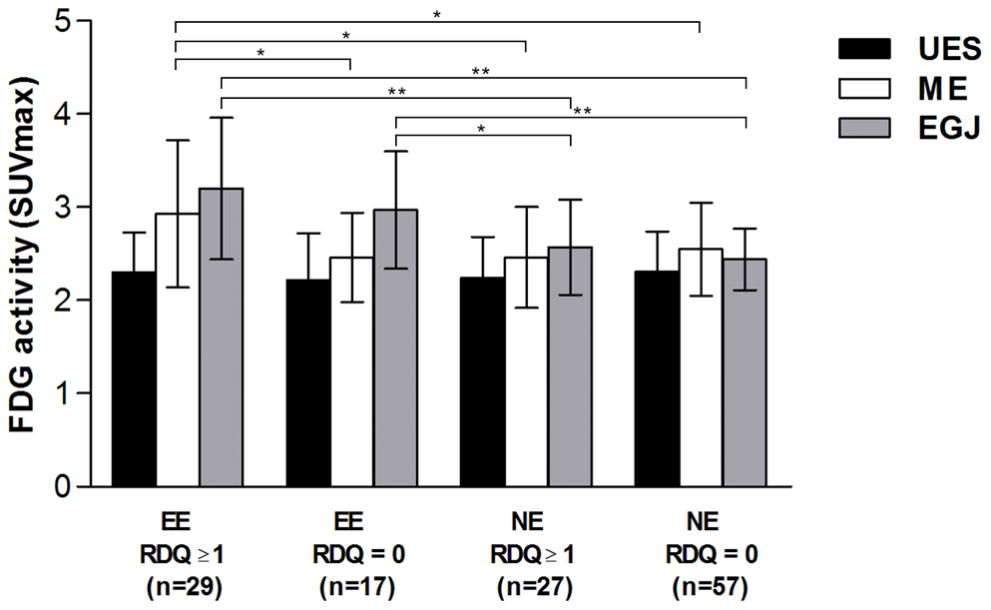
Comparison of SUV_max_ at index esophageal locations among 130 subjects with complete RDQ. Subjects were stratified by the presence of endoscopic erosive disease and gastroesophageal reflux symptoms. Abbreviation: FDG, ^18^F-Fluorodeoxyglucose; SUV_max_, maximum of standardized uptake values; EE, erosive esophagitis; NE, non-erosive; RDQ, Reflux Disease Questionnaire; UES, upper esophageal sphincter; ME, middle esophagus; EGJ, esophagogastric junction. Data are presented as mean ± standard deviation (*P<.05, **P<.01, Student *t* test).

We further evaluated the relationship between gastroesophageal reflux symptoms and esophageal inflammation at each index esophageal location from 130 subjects who have also fulfilled the RDQ since 2010. We found that heartburn subscale and total RDQ scores positively correlated with higher SUV_max_ in middle esophagus (*r* = .262, *P* = .003; *r* = .227, *P* = .009). We also compared SUV_max_ at each esophageal location in subjects stratified by the presence of erosive esophagitis and gastroesophageal reflux symptoms. As shown in Figure 3, symptomatic subjects with erosive esophagitis had significantly higher SUV_max_ in middle esophagus than those asymptomatic subjects (2.93±0.79 vs. 2.46±0.48, *P* = .016) and all subjects without erosive esophagitis. However, subjects with asymptomatic erosive esophagitis still have significantly higher SUV_max_ in esophagogastric junction than those without erosive esophagitis whether they were symptomatic or not (2.97±0.63 vs. 2.57±0.51 and 2.44±0.53, *P* = .001 and .027, respectively). There was no significant difference of SUV_max_ at all three esophageal locations for subjects without erosive esophagitis whether they were symptomatic or not.

### Determinants of Esophageal Inflammation

As shown in Table 3, univariate analyses confirmed several traditional risk factors were associated with esophageal inflammation in terms of SUV_max_ at each index esophageal locations, including age, male gender, alcohol consumption, and markers of general and central obesity. Using multivariate stepwise regression analyses, only total cholesterol level (*P* = .003) and subcutaneous adipose tissue (*P*<.001) were independently associated with higher SUV_max_ at upper esophageal sphincter, while alcohol drinking (*P* = .03) and BMI (*P*<.001) were associated with higher SUV_max_ at middle esophagus. Age (*P* = .027) and waist circumference (*P*<.001) were independently associated with higher SUV_max_ at esophagogastric junction.

**Table 3:** Determinants of esophageal inflammation (SUV_max_) at index esophageal locations.

	Correlation		Multivariate regression		
	r	P*	β	SE	P*
**Upper esophageal sphincter**					
BMI	.134	.004			
Waist circumference	.165	<.001			
Total cholesterol	.158	.001	.002	.001	.003
Total adipose tissue	.175	<.001			
Subcutaneous adipose tissue	.188	<.001	.002	.001	<.001
**Middle esophagus**					
Male gender	.125	.007			
Drinking	.110	.0018	.162	.074	.030
BMI	.266	<.001	.051	.001	<.001
Waist circumference	.245	<.001			
Total adipose tissue	.178	<.001			
Visceral adipose tissue	.148	.001			
Subcutaneous adipose tissue	.146	.002			
**Esophagogastric junction**					
Age	.131	.005	.008	.004	.027
BMI	.198	<.001			
Waist circumference	.236	<.001	.019	.004	<.001
Total adipose tissue	.182	<.001			
Visceral adipose tissue	.178	<.001			
Subcutaneous adipose tissue	.133	.004			

a. Multiple linear stepwise regression analysis was performed using the SUV_max_ as a dependent variable and the independent variables of those variables with significant correlation.

b. Abbreviation: BMI, body mass index; SUV_max_, maximum of standardized uptake values.

c. *P<0.05 indicates statistical significance.

## Discussion

This present study shows that esophageal inflammation, demonstrated as SUV_max_ on PET/CT, has good correlation with the presence and severity of erosive esophagitis. The typical symptom of heartburn, but not acid regurgitation, correlates well with increased SUV_max_ of the middle esophagus. We further confirmed that obesity markers, including BMI, waist circumference, visceral and subcutaneous adipose tissue volumes are associated with the development of erosive esophagitis and/or increased esophageal inflammation.

Increased uptake of FDG, especially in the distal third of the esophagus, has been reported in a number of esophageal diseases such as radiation esophagitis, erosive esophagitis, and Barrett's esophagus [22]–[26]. In an esophagoduodenal anastomosis rat model, dynamic FDG PET imaging was found to be a powerful tool in detecting reflux esophageal injury and carcinogenic progression from intestinal metaplasia to early adenocarcinoma [27]. However, human studies of PET/CT findings in subjects with GERD are still limited, and most are of small sample size or incidental findings from related studies. Recently, using FDG-PET, Tsai et al. have found a good correlation between the endoscopic severity of esophagitis and the degree of abnormal FDG uptake at distal esophagus in 408 subjects receiving health check-ups. However, symptomatology and precise localization of the abnormal uptake of FDG were not addressed in their study [26]. Our study utilized a validated GERD symptom questionnaire and anatomical imaging of PET/CT and demonstrated that endoscopically proven esophagitis was associated with increased FDG uptake in the whole esophagus, not just in the lower esophagus and esophagogastric junction, suggesting extensive esophageal involvement in subjects with GERD. A recent population-based study also demonstrated that endoscopically erosive esophageal disease, but not non-erosive counterpart, increased the risk of esophageal adenocarcinoma [28]. Whether our findings highlight the role of inflammation in the pathophysiology of esophagitis-Barrett's-adenocarcinoma sequence warrants further exploration.

Endoscopy enables the detection of minute mucosal changes and facilitates further pathological examination, and currently is the mainstay of evaluating patients with reflux symptoms [29]. However, the correlation of endoscopic findings with symptoms and therapeutic responses remains unsatisfactory and a great proportion of patients have no esophageal mucosal changes on endoscopic examination, so called non-erosive reflux disease [30]. In the present study, we found that heartburn subscale and total RDQ scores positively correlated with higher SUV_max_ in middle esophagus. It provides a link between inflammation and GERD symptoms, and is consistent with previous histopathological and endoscopic studies. Isomoto et al. demonstrated proinflammatory cytokines and inflammatory cells in esophageal biopsy specimens from patients with reflux symptoms, as well as from patients with esophagitis [31]. Magnified and image-enhanced endoscopy also revealed the presence of inflammatory changes in the macroscopically normal esophageal mucosa of reflux patients [29], [32], [33]. Furthermore, endoscopic ultrasound has demonstrated increased thickness and blood flow in the esophageal mucosa and submucosa, suggesting inflammation in the entire wall of the lower esophagus in both erosive and non-erosive reflux disease [34]. These and our findings may provide clues to explain the broad spectrum of manifestations and unpredictable therapeutic responses in patients with GERD [30]. While endoscopy, can only reveal mucosal changes of the esophagus, PET/CT may detect cellular metabolic activity beneath the mucosa, e.g., esophageal muscle layer, adventitia, and even the paraesophageal space or mediastinum, and thus would be useful in the comprehensive evaluation of inflammatory activity and follow-up of therapeutic responses in patients with gastroesophageal reflux disease.

In the present study, several erosive esophagitis subjects had markedly elevated SUV uptake at the esophagogastric junction with the highest up to 6. In addition, a range of SUV_max_ values of 2.8–5.6 were also found in the esophagogastric junction of subjects with Barrett's esophagus, which overlapped with the range of values found in esophageal cancer reported by other studies[21], [35], [36]. Yeung et al. reported a high sensitivity of 99% with the peak SUV between 3.6 and 46 in the evaluation of subjects with esophageal cancer, including both squamous cell carcinoma and adenocarcinoma [35]. Similarly, Ott et al. also reported a peak SUV of 5.2 to 50.3 in 52 patients with adenocarcinoma at the esophagogastric junction [36]. Roedl et al.[21] compared the esophageal FDG uptake on PET/CT scans in 36 patients with Barrett's esophagus or early malignant esophageal lesions with those of 66 patients benign esophageal disorders such as reflux esophagitis. Although endoscopic confirmation of reflux esophagitis was not available in their study, the intensity of PET/CT FDG activity in the esophagus was low to moderate (SUV_max_ ≤ 4) for 82% of subjects with benign lesions and for all 6 subjects with Barrett's esophagus, compared with predominantly moderate to high PET/CT FDG activity in early malignant lesions. The authors also found that higher scores of focality of FDG uptake may help to differentiate early malignant lesions from benign esophageal lesions. In the present study, nonetheless, no high-grade dysplasia or esophageal cancer was found. Although PET/CT has the advantage of its non-invasive nature and satisfactory correlation with erosive changes, the sensitivity and specificity may not be high enough to differentiate Barrett's esophagus and associated esophageal neoplasms from benign lesions. More evidence may be needed to prove the clinical utility of PET/CT in future studies.

Another important finding we demonstrated in the present study is that subjects with erosive esophagitis have significantly higher SUV_max_ in esophagogastric junction whether they were symptomatic or not. Asymptomatic erosive esophagitis is not uncommon in subjects undergoing a routine health check-up. Till now, the risk factors and natural history of asymptomatic esophagitis remain unclear [37]. In view of possible effects of chronic inflammation on the neoplasm formation or accelerated progression in subjects with GERD, further interventional studies to evaluate whether aggressive anti-inflammatory approaches, such as acid suppression therapy or dietary chemoprevention, can prevent the disease progression in subjects with asymptomatic esophagitis, would be of clinical importance.

Lines of epidemiologic evidence have shown a close association between obesity and GERD and related complications. Obesity, especially central obesity, could lead to changes in gastroesophageal anatomy and physiology, such as reduced lower esophageal sphincter pressure, hiatal hernia, increased frequency of transient lower esophageal sphincter relaxation, esophageal motor abnormalities, elevated intragastric pressure and disorders of gastric accommodations, all of which could promote the gastroesophageal reflux [38]. Moreover, VAT is biologically active and produces a variety of inflammatory mediators including interleukin-6, tumor necrosis factor-α and leptins, which may facilitate the development and progression of GERD and its related complications. In the present study, we not only confirmed that abdominal VAT is a strong risk factor of erosive esophagitis [39]–[41], but also showed a positive correlation between VAT volume and esophageal inflammation at both middle esophagus and esophagogastric junction. Multivariate analyses also found BMI and waist circumference to be independent determinants of esophageal inflammation at middle esophagus and esophagogastric junction respectively. Although the association between waist circumference and SUV_max_ at the esophagogastric junction but not at the middle esophagus could be explained by reflux damage limited to the junction and distal esophagus, the underlying mechanisms for why BMI would be associated with inflammation at middle esophagus but not esophagogastric junction remain unclear. Whether general obesity, represented by BMI, would aggravate the distal esophageal inflammation to extend proximally in subjects through other pathways other than cytokines or mechanical factors deserves further investigation. Moreover, the correlation between each risk factor and esophageal inflammation were relatively weak individually, which may partly reflect the complex nature and multifactorial pathophysiology of GERD. Therefore, active weight control through diet modification and regular exercise to reduce the impact of obesity, general or visceral, on the esophageal inflammation and its related neoplastic progression could not be overemphasized.

One strength of this study is the relatively large number of subjects with comprehensive clinical information regarding the traditional risk factors of GERD, especially including the abdominal visceral and subcutaneous adipose tissue as determined by the CT scan. Moreover, we included detailed upper GI endoscopy findings and systematic quantification of FDG uptake using PET/CT at each segment of the upper GI tract for advanced analyses. Still our study has several limitations. Our program was self-referred and self-funded, and we cannot exclude the possibility that our participants might not readily represent a general population and selection bias might exist. Many subjects were asymptomatic and the case number of subjects with high grade erosive esophagitis and/or Barrett's esophagus was also relatively small, thus we may not have adequate power to address the interplay between symptomatology, endoscopically evident mucosa damage and SUV_max_ on PET/CT. Further prospective studies to enroll patients with high grade esophagitis, Barrett's esophagus, esophageal adenocarcinoma or other inflammatory disorders, such as eosinophilic esophagitis, fungal or viral infection in large scale may provide more insights into the pathophysiology of the esophagitis to adenocarcinoma sequence. Second, the actual involvement extent of erosive change in the distal esophagus is difficult to be ascertained with current endoscopic classification method and this may impact on the SUV_max_ measured at the more proximal esophageal locations. Furthermore, ambulatory pH monitoring was not used in the present study and thus the diagnosis of non-erosive reflux disease could not be reliably made. Further studies incorporating the novel combined pH-impedance monitoring may help to clarify the complex relationship between the nature of refluxate (acidic, weakly acidic or non-acidic), reflux extent, reflux duration, associated symptoms, and esophageal inflammation. Third, besides partial volume and misregistration between the PET and CT which may lead to less accurate SUV measurement in small lesions, diluted barium solution or water to distend and demarcate the stomach and bowels was not used in our routine FDG PET/CT imaging protocol, which might cause false-positive results in the stomach and duodenum [42]. Although SUV based on the total body weight is the most commonly used method nowadays, it might be overestimated in obese individuals [43]. In addition, the glucose level, diabetes mellitus, insulin treatment and obesity may affect FDG biodistribution and SUV measurements and thus limit the clinical application of the present study [44]. Finally, besides the concern of radiation exposure, the cost and benefit of FDG PET/CT should be carefully balanced in the current economy of escalating health costs and utilization disparity. Whether our pilot findings could be readily translated into clinical practice deserves further economic evaluation.

In conclusion, our study demonstrated that esophageal inflammation as shown by FDG using PET/CT correlates well with the endoscopic severity and symptomatology of GERD. Moreover, obesity markers, including BMI, waist circumference, visceral and subcutaneous adipose tissue, are associated with the increased esophageal inflammation and related complications. With the ever-increasing prevalence of GERD and obesity, further prospective studies focusing on the evolution of esophageal inflammation during acid suppression treatment and surveillance of Barrett's esophagus and related malignant transformation in patients with chronic reflux disease are warranted.
